# Probiotic-Mediated Inhibition of Skin Pathogens and Modulation of Innate Immune Responses in HaCaT Cells

**DOI:** 10.3390/microorganisms14081754

**Published:** 2026-08-10

**Authors:** Maria Magdalena Coman, Lucia Occhigrossi, Stefania Silvi, Giulia Nannini, Amedeo Amedei, Valerio Napolioni, Maria Cristina Verdenelli

**Affiliations:** 1 Synbiotec S.r.l., Spin-off of UNICAM, Via Gentile III Da Varano, 62032 Camerino, Italy; cristina.verdenelli@unicam.it; 2 School of Advanced Studies, University of Camerino, 62032 Camerino, Italy; lucia.occhigrossi@unicam.it; 3 School of Biosciences and Veterinary Medicine, University of Camerino, 62032 Camerino, Italy; stefania.silvi@unicam.it (S.S.); valerio.napolioni@unicam.it (V.N.); 4 Department of Experimental and Clinical Medicine, University of Florence, 50134 Florence, Italy; giulia.nannini@unifi.it (G.N.); amedeo.amedei@unifi.it (A.A.)

**Keywords:** probiotics, acne, skin inflammation, skin pathogens, skin care, HaCaT keratinocytes, cytokines

## Abstract

*Acne vulgaris* is a highly prevalent inflammatory skin condition involving the interaction between skin microbes and host immunity, which lead with the changes in composition and activities of the skin microbiota disturbing its homeostasis. Several probiotic strains have been tested for their anti-pathogenic activity against the main acne-responsible bacteria, their capacity to contrast pathogenic adhesion to HaCaT cells and the in vitro down-regulation of innate immunity. To investigate whether probiotics have direct effects on the growth of *Cutibacterium acnes*, *Staphyloccoccus aureus*, *Streptococcus pyogenes* and *Streptococcus epidermidis*, the antimicrobial activity of six probiotics was analyzed by two different methods (modified cross-streak and agar well diffusion). The in vitro blockage of pathogens’ adherence by the probiotic strains to HaCaT cells was also investigated, under three possible mechanisms: exclusion by adhered probiotics, displacement of adhered pathogens and competition for receptor sites. The inflammatory response was evaluated by the Luminex approach, targeting a selection of innate immune markers in the HaCaT cell culture media. All probiotic strains showed anti-pathogenic activity against the pathogens tested. The inhibition result on HaCaT cells highlights a significant (*p* < 0.05) competition of all probiotics against all pathogens. Each pathogenic strain alone led to an up-regulation of innate immune markers, while restoration of the microbiome diversity by probiotics presence may suppress inflammation via down-regulation of innate immunity. The results suggest that the tested probiotics could prevent colonization of the skin by relevant pathogens through barrier and interference mechanisms (mainly exclusion), suggesting a potential use in the future conventional therapies of skin disorders.

## 1. Introduction

*Acne vulgaris* is an inflammatory, multifactorial disease that increasingly affects adults and, beyond its physical manifestations, exerts a significant psychological impact, disrupting individuals’ daily social lives [1,2,3]. The term microbiota refers to the community of living microorganisms (including bacteria, archaea, viruses and eukaryotic microbes) inhabiting a specific environment (such as the gut and the skin) [4]. More than 40 bacterial genera have been identified on human skin, mainly belonging to four phyla: Actinobacteria, Firmicutes, Proteobacteria and Bacteroidetes [5,6]. Existing research emphasizes the vital roles of commensal bacteria on our skin as they provide essential protection against pathogens by competing for resources and space and produce antimicrobial substances like bacteriocins [5,6]. Moreover there is a significant connection between skin microbiota and the innate immune system as microbes activate Toll Like Receptors 2 (TLR2), leading to the down-regulation of inflammatory cytokines, and improve tight junctions, enhancing the barrier function [7].

Usually describing the pathogenesis of *Acne vulgaris* are four main causes depicted, namely altered bacterial colonization, inflammation, increased sebum production and keratinocyte hyperproliferation [8,9]. The disease onset is characterized by an increased production of sebum by atrophic sebaceous glands and by the formation of comedones [10]. Comedogenesis is the consequence of the accumulation of corneocytes in the pilosebaceous duct due to the contact with comedogenics factors such as anomalies of the sebaceous lipids, or cytokines as interleukin (IL)-1α [8,9,11]. The right chronology of events that lead to acne outcome still has to be clarified, but the latest research assumes that inflammation may begin at a very early stage of acne’s lesion (microcomedones) development, even before the primary hyperproliferative changes [12]. Interestingly, inflammatory diseases have also been frequently associated with dysbiosis, and this inflammatory status is currently being investigated as a potential mediator linking dysbiosis to skin disorders [13]. Some studies suggest there is an increased presence of gut bacterial DNA in the bloodstream of individuals with chronic skin conditions, which is attributed to the communication of the intestinal and epidermal barriers through systemic circulation (blood and lymph) [5].

*Cutibacterium acnes* is indeed the prevalent microorganism in the pilosebaceous unit. It is present on the skin in physiological conditions such as commensal, playing a relevant role in protection from the pathogens, keeping the natural pH of the skin and sebaceous glands balanced, through triglycerides’ hydrolyzation, releasing free fatty acids and producing propionic acid [8,14]. Eventually, it can act as a pathogen involved in pathogenesis of *Acne vulgaris*, interacting with the innate immunity (Toll-like receptors, antimicrobial peptides, protease-activated receptors and matrix metalloproteinase) and increasing the production of pro-inflammatory cytokines by human keratinocytes, sebocytes and macrophages [15]. Additionally, when *C. acnes* releases extracellular vesicles, it triggers an acne-like pattern, causing keratinocytes overgrowth and altering their differentiation, impacting the expression of skin markers [16]. The association between *C. acnes* and acne pathogenesis is undeniable; what still needs clarification is the manner and timing of the transition of this commensal bacterium to a pathogenic state. Recent studies are questioning whether the role of *S. epidermidis* may also be pivotal in the disease pathogenesis, suggesting that acne may arise from an imbalanced relationship between these two microorganisms (*C. acnes* and *S. epidermidis*), which is normally symbiotic, but potentially harmful [17]. *S. aureus* is the most common nosocomial, opportunistic pathogen, with mortality rates ranging from 6% to 40% [18]. In inflammatory skin diseases, it grows significantly and produces a series of virulence factors aggravating the inflammatory responses, mainly through the production of V8 serine protease and the formation of biofilms which hinders the efficacy of antibiotics by shielding pathogenic bacteria from host immune cells like neutrophils and macrophages [19]. *S. pyogenes* is widely recognized as one of the most prevalent pathogens affecting the skin, contributing to various skin disorders such as impetigo and ecthyma or even more debilitating conditions like necrotizing fasciitis [20,21]. While research on its involvement in acne pathogenesis is limited, its presence on the skin is associated with an imbalanced state so that targeting *S. pyogenes*, along with other pathogens, may offer a promising therapeutic approach to restore skin equilibrium and aid in the healing of acne.

Building on the insights into acne pathogenesis, current acne therapies are bifocal employing both topical and systemic (oral drugs) approaches which focus on *preventing* the onset of future lesions on one side and healing existing ones on the other side. Systemic therapies address the root causes such as inflammation (antibiotics), hormonal imbalance (antiandrogens) or dysbiosis (probiotics and prebiotics) to enhance overall acne management. Considering the significance of maintaining a balanced homeostasis, as previously discussed, it is crucial to note that systemic antibiotics aimed at reducing *C. acnes* could potentially disrupt this balance. This disruption might lead to opportunistic infections in the hair follicles caused by competing species, such as *Pseudomonas* ones [22]. However, the primary concern with antibiotic therapy remains the development of antibiotic resistance [22]. The alternative therapeutic avenue involves topical treatments, which have the advantage of exposing more of the pilosebaceous units to the treatment, but at the same time, as a side effect, this could lead to skin irritation risk [23]. Overall, current therapies frequently exhibit a spectrum of side effects, ranging from mild to significant, potentially compromising both patient compliance and therapeutic effectiveness. Hence, the escalating intrigue in the role of probiotics and prebiotics is noteworthy [24]. Probiotics and prebiotics can be used in the modulation of skin microbiota (topically) or of gut microbiota (systemically), supporting the traditional therapies and ameliorate the healing process, affecting either the pathogenesis of acne itself, or the psychological component of this disease (by the gut–brain axis). Oral consumption of prebiotics and probiotics has shown to decrease systemic markers of inflammation and oxidative stress present in acne disease with interesting studies supporting the use of topical treatments too [7,25,26,27,28].

The present study aims to evaluate the capacity of six probiotic strains (previously characterized) to compete with pathogens implicated in acne disorder according to the current literature. The probiotics’ ability to prevent pathogen adhesion and displace pathogens already adhered to keratinocytes was assessed thereby simulating potential applications before and after disease onset. The antimicrobial activity, the interaction with the immune system and the effects on cytokine production of the six strains were investigated with the final aim to evaluate their potential use in skin condition treatments.

## 2. Materials and Methods

### 2.1. Bacterial Strains and Culture Conditions

The probiotic strains used, namely, *Lacticaseibacillus rhamnosus* IMC 501^®^ and 513, *Lacticaseibacillus paracasei* IMC 502^®^, *Lactiplantibacillus plantarum* IMC 509 and 301, *Lactococcus lactis* IMC 533, were provided by Synbiotec S.r.l. (Camerino, Italy). The strains, listed in Table 1, were isolated from human subjects within the European project CROWNALIFE and described for their main probiotic characteristics [29,30,31,32].

Standard pathogenic bacteria, key players in acne development, *Cutibacterium acnes* DSM 1897, *Staphylococcus aureus* ATCC 25923, *Staphylococcus epidermidis* DSM 20044 and *Streptococcus pyogenes* DSM 2071 (Table 1), were purchased from ATCC and DSM (American Type Culture Collection, Manassas, Virginia and Deutsche Sammlung von Mikroorganismen und Zellkulturen GmbH, Braunschweig, Germany).

All the strains, once activated, were maintained at −80 °C in 15% (*w*/*w*) glycerol. De Man Rogosa and Sharpe (MRS) broth (Oxoid Ltd., Basingstoke, Hampshire, UK) for lactobacilli and Tryptic Soy Broth (TSB, Oxoid, Basingstoke, Hampshire, UK) for pathogens strains were inoculated from the stock culture collection and incubated for 24–48 h at 37 °C under aerobic/anaerobic conditions.

### 2.2. Antimicrobial Activity Assessment

In order to evaluate the antimicrobial activity of the six probiotic strains, looking also to the mechanistic aspect, two methods were applied: (a) the agar well diffusion assay [33] and (b) a modified radial streak method [33].

#### 2.2.1. Agar Well Diffusion Assay

Each pathogen strain was cultivated at the proper optimal conditions (TSA, 48 h, 37 °C) and a bacterial cell suspension of 0.5 McFarland standard (corresponding to 1.5 × 10^8^ CFU/mL) was prepared in physiological solution. Modified 1:1 MRS/TSA agar plates were spread with each of the indicator pathogen strains previously prepared.

In parallel, each probiotic strain was cultivated in MRS broth for 24 h at 37 °C and cell-free supernatant (CFS) was obtained by centrifuging each culture at 13,000 rpm for 20 min. Six wells of 10 mm of diameter were cut into the pathogen-spread agar plates with a sterile metal cylinder, and then 50 μL of each CFS was placed into each well. A negative control, the uninoculated culture medium, was processed under the same conditions as the probiotic cultures and tested in parallel.

The plates were incubated for 24 h at 37 °C and antimicrobial activity recorded as growth-free inhibition zones around the wells. Inhibition zones were measured in mm from the edge of the wells.

#### 2.2.2. Radial Streak Method

Modified MRS/TSA agar plates were prepared and inoculated with 0.5 McFarland (1.5 × 10^8^ CFU/mL) of each probiotic bacterial suspension by covering a circular agar surface in the center of the Petri dish. After 48 h of incubation at 37 °C, the plates were spread with pathogen indicator strains (0.5 McF) by radial lines of inoculum from the border to the center of the plate. The microbial interactions were analyzed after 24 h of incubation at 37 °C by the observation of the inhibition zone size. The inhibition zone diameter (IZD, cm) was subtracted from the circle diameter (CD, cm) of the probiotic strain spreading zone to determine the growth inhibitory activity (GI). GI is equal to (IZD-CD)/2 [34].

### 2.3. Adhesion Capacity Assessment

To study the adhesion ability of the probiotic strains, an adhesion assay was performed using the HaCaT cells as the skin model.

#### 2.3.1. Cell Lines and Viability

HaCaT cells, spontaneously immortalized human keratinocyte line, were kindly provided by Cell Line Service GmbH (Eppelheim, Germany). First, HaCaT keratinocytes were seeded at a density of 1 × 10^5^ cells/well in 96-well plates and cultured for 24 h in Delbecco’s Minimal Essential Medium (DMEM, Sial Srl, Rome, Italy) containing 10% fetal bovine serum (FBS), 1% L-glutamine and 1% antibiotic/antimycotic. To evaluate a possible cell toxicity, each probiotic strain was tested at four different bacterial loads: 1 × 10^7^, 1 × 10^8^, 1 × 10^9^ and 1 × 10^10^ CFU/mL. All probiotic-treated cells were incubated in a CO_2_-incubator at 37°C for 24 h. The cell viability of each treatment was determined using the MTT (3-(4,5-dimethylthiazol-2-yl)-2,5-diphenyltetrazolium bromide) assay, a colorimetric assay for reflecting the number of viable cells present [35]. The absorbance of the sample against a background control was measured at 570 nm wavelength using an ELISA reader microliter plate (BioTek Instruments, Winooski, VT, USA).

#### 2.3.2. Adhesion Assay

Adhesion assay was performed using a 6-well culture plate containing a sterile coverslip in each well. Each well was then filled with 2 mL of HaCaT cell suspension at a concentration of 1 × 10^5^ cells/mL and incubated in a 5% CO_2_ atmosphere at 37 °C. After 48 h, when the HaCaT cells were grown to approximately 60% confluence, they were washed twice with PBS and inoculated with 2 mL of bacteria suspension. The assay was performed either using lactobacilli or pathogenic suspension at a concentration of 1.5 × 10^9^ CFU/mL. The bacterial cells were washed and resuspended in a modified medium (mDMEM, DMEM supplemented with 5% of bacterial broth, MRS for the probiotic bacteria or TSB for the pathogenic bacteria) immediately prior co-incubation in order to prevent the fast acidification or nutrient depletion typically caused by standard bacterial media. The plates were then incubated for 1 h at 37 °C in a 5% CO_2_ atmosphere to allow the adhesion of bacteria on HaCaT cells. At the end, the cells were washed, Gram stained and observed under the microscope (1009; Leitz Laborlux 12 microscope, Ernst Leitz Wetzlar GmbH, Wetzlar, Germany) for confirming the presence and counting the number of bacteria attached to each cell. Each adherence assay was conducted in duplicate, repeated in three independent experiments and 50 randomly chosen cells were evaluated for microorganism adhesion. Moreover, for each single test, 20 different microscope fields were examined, and on each field the number of HaCaT cells and the total number of adhered bacterial cells was calculated. Therefore, the microscopy-based parameters (bacterial counts and adhesion index) describe the distribution and extent of bacterial attachment on individual epithelial cells, whereas the CFU-based adhesion percentage quantifies the fraction of viable bacteria able to adhere. The combined use of these complementary endpoints provides a rightly-comprehensive assessment of probiotic adhesive capacity by integrating both cellular-level interactions and overall bacterial retention.

### 2.4. Competition Capacity Assessment

Competition assays, especially in skin-relevant models like HaCaT keratinocytes, offer supplementary mechanistic insight into how probiotics interact with pathogens at the epithelial interface. These assays evaluate several ecological interactions that replicate actual host–microbiome dynamics by combining exclusion (pre-colonization), displacement (post-adhesion), and inhibition (co-incubation) approaches. In a preventative colonization scenario modeled by exclusion assay, probiotics initially occupy keratinocyte binding sites and form a physical and metabolic barrier against incoming pathogenic bacteria, while the displacement approach assesses probiotics’ capacity to reduce/eliminate pathogens which have already attached to HaCaT cells, indicating a therapeutic rather than preventative strategy. Inhibition assay mimics early-stage microbial interactions on the skin’s surface, when commensals, probiotics and pathogens vie for scarce epithelial receptors and nutrients.

Three types of assay were performed to study the ability of lactobacilli to interfere with the adherence of pathogenic strains to HaCaT cells: exclusion, displacement and inhibition assay [32,36].

#### 2.4.1. Exclusion Assay

The exclusion assay was performed using 6-well culture plates containing a sterile coverslip in each well. In each plate, the wells were prepared and incubated to allow for the bacteria adhesion on the HaCaT cells, as described before.

After 1 h, the cells were washed twice with PBS to remove all non-adhering probiotic bacteria and then inoculated with pathogenic strains following the same experimental conditions (1 mL at a concentration of 1.5 × 10^9^ CFU/mL). At the end, the cells were washed, Gram stained and observed under the microscope (1009; Leitz Laborlux 12 microscope, Ernst Leitz Wetzlar GmbH, Wetzlar, Germany). Further, each HaCaT cell was scored for the presence and number of bacteria attached, either pathogenic or lactobacilli, morphologically recognizable and distinguishable. Each exclusion assay was conducted in duplicate and 50 randomly chosen cells were evaluated for microorganism adhesion. Moreover, 20 different microscope fields were examined for each slide, and on each field, the number of HaCaT cells and the total number of adhered bacterial cells was calculated.

#### 2.4.2. Displacement Assay

The displacement test evaluates the ability of the lactobacilli to displace the already adhered pathogens and was performed with the same protocols of the exclusion test but with a reversed inoculation sequence, which implies that the HaCaT cells were inoculated first with pathogenic strains and then with lactobacilli.

#### 2.4.3. Inhibition Assay

In the inhibition test, the HaCaT cells were incubated with both probiotic and pathogenic strains at the same time, for 1 h at 37 °C, in 5% of CO_2_ atmosphere. This specific assay aimed to evaluate the capacity of both types of bacteria (probiotics and pathogens) to compete for the adhesion to the same HaCaT cells and at the same time to also evaluate the ability of each probiotic strain to reduce the adhesion of the pathogenic ones.

### 2.5. Immune Biomarkers In Vitro Modulation

The inflammatory response was evaluated in culture media of the treated HaCaT cells through the test of 7 cytokines by Milliplex Map Human cytokine/chemokine/growth factor panel A and TGFβ1 single plex magnetic bead kits (Merck KGaA, Darmstadt, Germany), following the manufacturers’ instructions. In detail, interferon (IFN)-γ, interleukin (IL)-10, IL-4, IL-6, IL-8, IL-17A and TGFβ1 were detected. Regarding the Assay Sensitivity, the minimum detectable concentrations (MinDC) for the evaluated cytokines are reported in Table 2. Plates were read on a Luminex MagPix instrument. The levels of cytokines were estimated using a 5-parameter polynomial curve (Bio-plex Manager 6.2 Software, Biorad Laboratories, Hercules, CA, USA).

### 2.6. Statistical Analysis

All experiments were performed in three independent biological replicates and each measurement was analyzed in duplicate. Data are presented as mean ± standard deviation (SD) or standard error of the mean (SEM), as indicated.

To evaluate the combined effects of probiotic strain, pathogen species and competition assay (exclusion, displacement and inhibition), pathogen adhesion data were analyzed using a full factorial model including all main effects and interaction terms. Experimental replicate was included as a blocking factor to account for variability among independent experiments. For each fixed effect, F statistics, degrees of freedom, *p* values, partial eta-squared (η^2^p) effect sizes and corresponding 95% confidence intervals were calculated. The statistical analyses were performed using SPSS 27.0 (SPSS Inc., Chicago, IL, USA).

Whenever significant interactions were detected, post hoc comparisons among probiotic strains were performed separately within each pathogen–assay combination using one-way ANOVA followed by Tukey’s multiple comparison test, using GraphPad PRISM^®^ 8.4.2 program (GraphPad software, San Diego, CA, USA), allowing biologically meaningful interpretation of strain-specific effects under comparable experimental conditions. Statistical significance was established at *p* < 0.05.

## 3. Results

### 3.1. Antipathogenic Activity Assessment

#### 3.1.1. Agar Well Diffusion Assay

The inhibition areas regarding each pathogen tested were measured for each cell-free supernatant (CFS) from each probiotic strain culture; the means and the standard deviations were then calculated (Table 3).

The agar well diffusion test had the aim to evaluate whether the inhibition was dependent on metabolites of probiotic strains. All the CFS of probiotic strains showed a high inhibitory activity against the pathogenic strains, even though *L. lactis* IMC 533 was the one with the weakest effect. The most susceptible pathogen resulted with *S. pyogenes* DSM 2071, with the highest inhibition zone of 21.6 mm when in contact with CFS of *L. rhamnosus* IMC 501^®^.

*C. acnes* DSM 1897 resulted with being the one less inhibited compared to the other pathogenic strains, but with high values for the inhibition zones.

#### 3.1.2. Radial Streak Method

With this method, the inhibition areas regarding the tested pathogens were measured versus each probiotic strain and the means and the standard deviations are shown in Table 4.

The radial streak testing documented distinct, strain-dependent antimicrobial activity of the probiotic strains against all four skin-associated pathogenic bacteria, with inhibition widths varying from modest (≈2 mm) to high (>20 mm), representing synthesis of diffusible different antagonistic substances.

Both strains of *L. rhamnosus* (IMC 501^®^ and 513) exhibit the most reliable and broad- antimicrobial spectrum, combining potent inhibition against *S. pyogenes* DSM 2071, *S. aureus* ATCC 25923, and *C. acnes* DSM 1897. The same behavior may also be observed for the two *L. plantarum* strains (IMC 509 and 301), while strains *L. paracasei* IMC 502^®^ and *L. lactis* IMC 533 have the lowest overall inhibitory power within the studied pathogenic species.

### 3.2. Adhesion Capacity

#### 3.2.1. Cell Viability by MTT Assay

**Figure 1:**
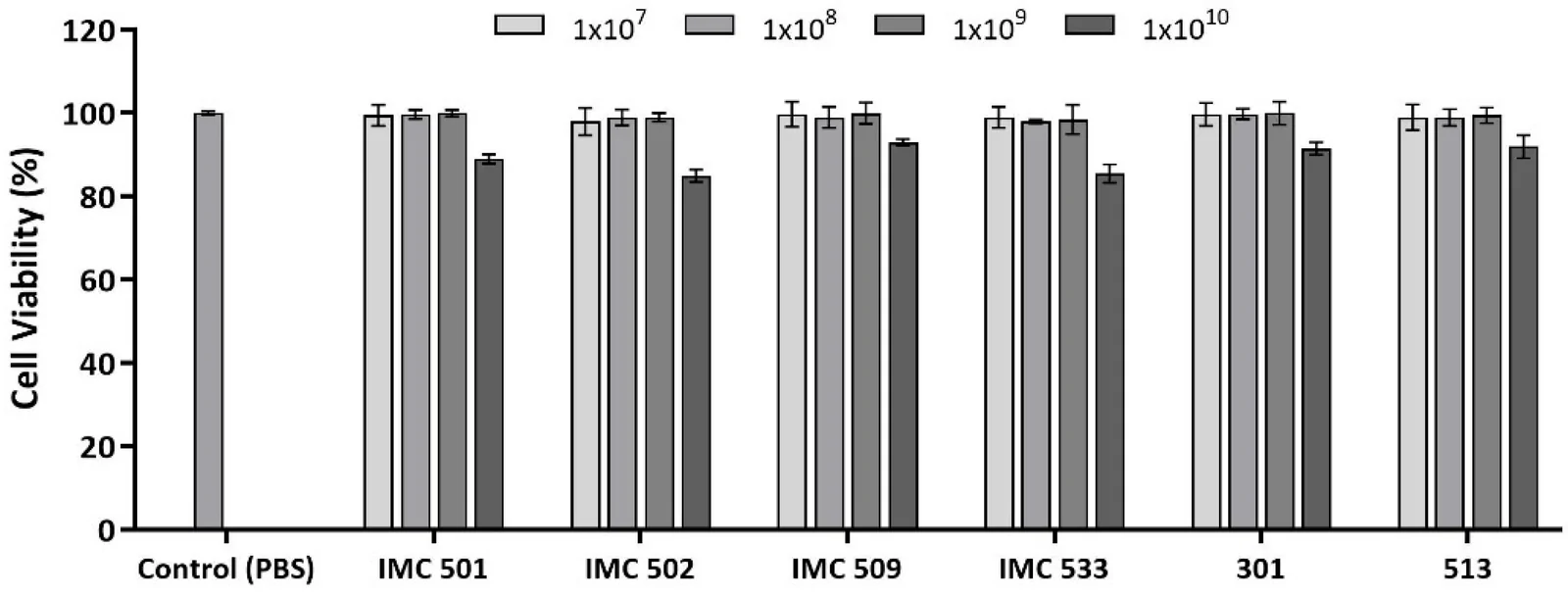
Probiotic cytotoxicity on HaCaT cells. The probiotic strains IMC 501 (*L. rhamnosus* IMC 501^®^), IMC 502 (*L. paracasei* IMC 502^®^), IMC 509 (*L. plantarum* IMC 509), IMC 533 (*L. lactis* IMC 533), 301 (*L. plantarum* 301) and 513 (*L. rhamnosus* 513) were added at different bacterial loads (from 10^7^ to 10^10^ CFU/mL) and cell viability was evaluated by MTT assay. The data are representative of three separated experiments and expressed as mean ± SEM.

The cell viability test was performed to completely exclude the potential cytotoxicity of probiotics on HaCaT cells. The MTT assay showed that probiotics were not cytotoxic to HaCaT cells (Figure 1), and the bacterial loads between 10^8^ to 10^9^ CFU/mL were selected for the next experiments. The bacterial load of 10^10^ CFU/mL was excluded since an excess of probiotic debris was found in the wells interfering with the cell viability and assay.

#### 3.2.2. Adhesion Assay

The adherence capability of probiotic and pathogen strains to HaCaT cells is shown in Table 5. It is expressed as adhesion percentage, average of the number of adhered microorganisms per cell and adhesion index.

*L. plantarum* 301 showed the highest adhesion percentage (11.1%), followed by *L. plantarum* IMC 509 (6.5%), while all the others displayed an adhesion percentage ranged between 3.4 and 4.5%. Pathogens adhered to HaCaT cells with a lower adhesion percentage compared to probiotic bacteria. *S. pyogenes* DSM 2071 showed the highest adhesion percentage (3.3%), whereas *C. acnes* DSM 1897 was the one with the lowest (0.9%). The adhesion capacity of the different bacterial strains to HaCaT cells was expressed both as percentage and average of the number of adhered microorganisms per cell. Most of the data obtained from microscopic observation confirmed the results of the bacterial viable count, whereas in a few cases, a discrepancy between the results of the two tests was detected, such as for *L. lactis* IMC 533.

Among probiotic strains, both *L. plantarum* IMC 509 and 301 exhibited the highest Adhesion Index (AI), with values of 4212 and 4160, respectively, indicating strong interaction with the HaCaT cell surface. In particular, *L. lactis* IMC 533 showed the highest number of adherent bacteria per cell (98.3 ± 47.0) and highest AI of 4467, despite a relatively low adhesion percentage (3.4%), suggesting highly efficient localized surface aggregation. On the other hand, the pathogenic strains also displayed strong adhesive capacity, with an AI of 4694 and 92.2 ± 42.1 adherent bacteria per HaCaT cell in the case of *S. pyogenes* DSM 2071, for example. *S. aureus* ATCC 25923 also showed substantial adhesion capacity (AI = 3510), whereas *S. epidermidis* DSM 20044 exhibited intermediate adherence (AI = 2204). In contrast, *C. acnes* DSM 1897 demonstrated the lowest adhesion ability, with an adhesion percentage below 1% and an AI of only 238, indicating limited direct attachment to keratinocytes under the tested conditions.

### 3.3. Competition Assessment

Before analyzing the individual competition assays, pathogen adhesion data were evaluated using a factorial model including probiotic strain, pathogen species, competition assay and their interactions. Significant main effects of strain, pathogen and assay were observed (all *p* < 0.001). Moreover, significant strain × pathogen, strain × assay, pathogen × assay and three-way strain × pathogen × assay interactions were detected (Supplementary Table S1), indicating that the inhibitory activity of the probiotic strains depends on the specific pathogen and on the competition mechanism employed.

The three-way interaction showed a very large effect size (partial η^2^ = 0.857, 95% CI 0.814–0.880), demonstrating that the biological response cannot be explained by any single experimental factor alone. Therefore, the following analyses are presented separately for each pathogen–assay combination to facilitate biological interpretation of these significant interactions.

#### 3.3.1. Exclusion Assay

**Figure 2:**
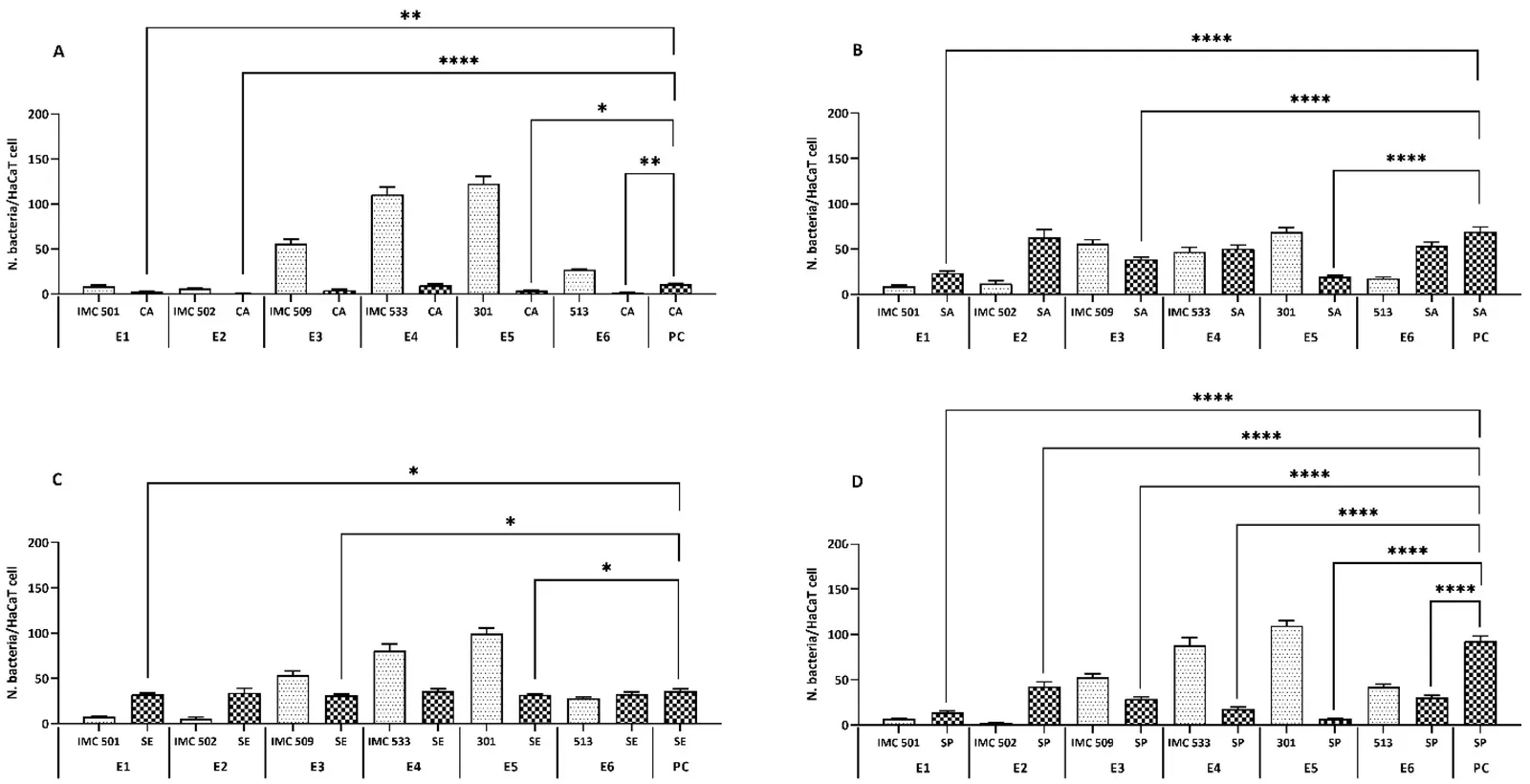
Inhibitory effect of probiotic bacteria (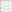) on pathogen (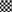) adhesion to HaCaT cells detected by exclusion assay (E1–E6). HaCaT cells were incubated with six probiotic strains (IMC 501—*L. rhamnosus* IMC 501^®^, IMC 502—*L. paracasei* IMC 502^®^, IMC 509—*L. plantarum* IMC 509, IMC 533—*L. lactis* IMC 533, 301—*L. plantarum* 301, 513—*L. rhamnosus* 513) and then infected with CA—*Cutibacterium acnes* DSM 1897 (**A**), SP—*Streptococcus pyogenes* DSM 2071 (**B**), SE—*Streptococcus epidermidis* DSM 20044 (**C**) or SA—*Staphylococcus aureus* ATCC 25923 (**D**). Panel E1–E6 represent the six exclusion assay conditions: E1—IMC 501 vs. each pathogenic strain, E2—IMC 502 vs. each pathogenic strain, E3—IMC 509 vs. each pathogenic strain, E4—IMC 533 vs. each pathogenic strain, E5—301 vs. each pathogenic strain and E6—513 vs. each pathogenic strain. PC indicates positive control and represents the adhesion of each single pathogen. Results are expressed as mean ± SEM. Significance levels are indicated as * *p* < 0.05, ** *p* < 0.01, **** *p* < 0.0001 using one-way ANOVA followed by multiple comparison tests.

The exclusion assay revealed that the adherence capacity of each pathogenic strain tested was considerably decreased by the pre-colonization of HaCaT cells by the probiotic strains (Figure 2), evidencing a selective antipathogen exclusion rather than non-specific antimicrobial activity.

In detail, all probiotic strains significantly reduced the adhesion of *S. pyogenes* DSM 2071, *C. acnes* DSM 1897, *S. aureus* ATCC 25923 and *S. epidermidis* DSM 20044, except for IMC 533, which did not show excessive suppression of *C. acnes* DSM 1897 and *S. epidermidis* DSM 20044. The strongest inhibition of all acne-associated pathogens was observed by the activity of IMC 501^®^, IMC 509, 301 and 513.

#### 3.3.2. Displacement Assay

**Figure 3:**
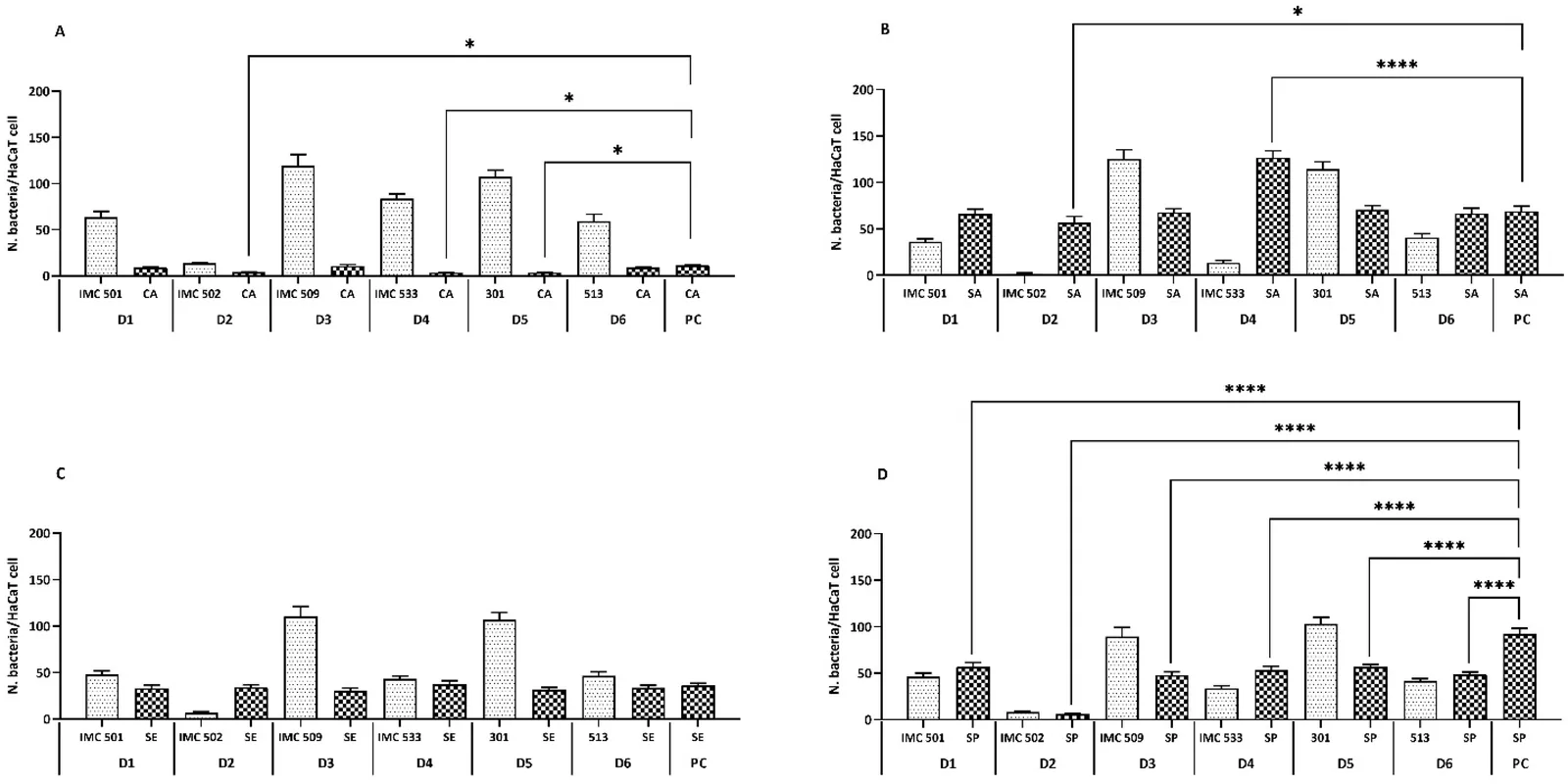
Inhibitory effect of probiotic bacteria (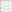) on pathogen (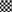) adhesion to HaCaT cells detected by displacement assay (D1–D6). HaCaT cells were incubated with six probiotic strains (IMC 501—*L. rhamnosus* IMC 501^®^, IMC 502—*L. paracasei* IMC 502^®^, IMC 509—*L. plantarum* IMC 509, IMC 533—*L. lactis* IMC 533, 301—*L. plantarum* 301, 513—*L. rhamnosus* 513) after infection with CA—*Cutibacterium acnes* DSM 1897 (**A**), SP—*Streptococcus pyogenes* DSM 2071 (**B**), SE—*Streptococcus epidermidis* DSM 20044 (**C**) or SA—*Staphylococcus aureus* ATCC 25923 (**D**). Panel D1–D6 represent the six exclusion assay conditions: D1—IMC 501 vs. each pathogenic strain, D2—IMC 502 vs. each pathogenic strain, D3—IMC 509 vs. each pathogenic strain, D4—IMC 533 vs. each pathogenic strain, D5—301 vs. each pathogenic strain and D6—513 vs. each pathogenic strain. PC indicates positive control and represents the adhesion of each single pathogen. Results are expressed as mean ± SEM. Significance levels are indicated as * *p* < 0.05, **** *p* < 0.0001 using one-way ANOVA followed by multiple comparison tests.

Compared to the exclusion, displacement assay showed a strain-dependent and generally slightly limited anti-adhesion effect, indicating that probiotics are less effective in removing already-adhered pathogens than at preventing primary colonization (Figure 3).

The probiotic strains IMC 502^®^ and IMC 533 significantly reduced the already-adhered *C. acnes* DSM 1897, *S. pyogenes* DSM 2071 and *S. aureus* ATCC 25923 in comparison to the single pathogen (positive controls). On the other hand, probiotics were less effective on *S. epidermidis* DSM 20044 removal, indicating limited displacement efficacy against commensal bacteria, which is ecologically advantageous and shows microbiome compatibility rather than broad-spectrum competitive elimination.

#### 3.3.3. Inhibition Assay

**Figure 4:**
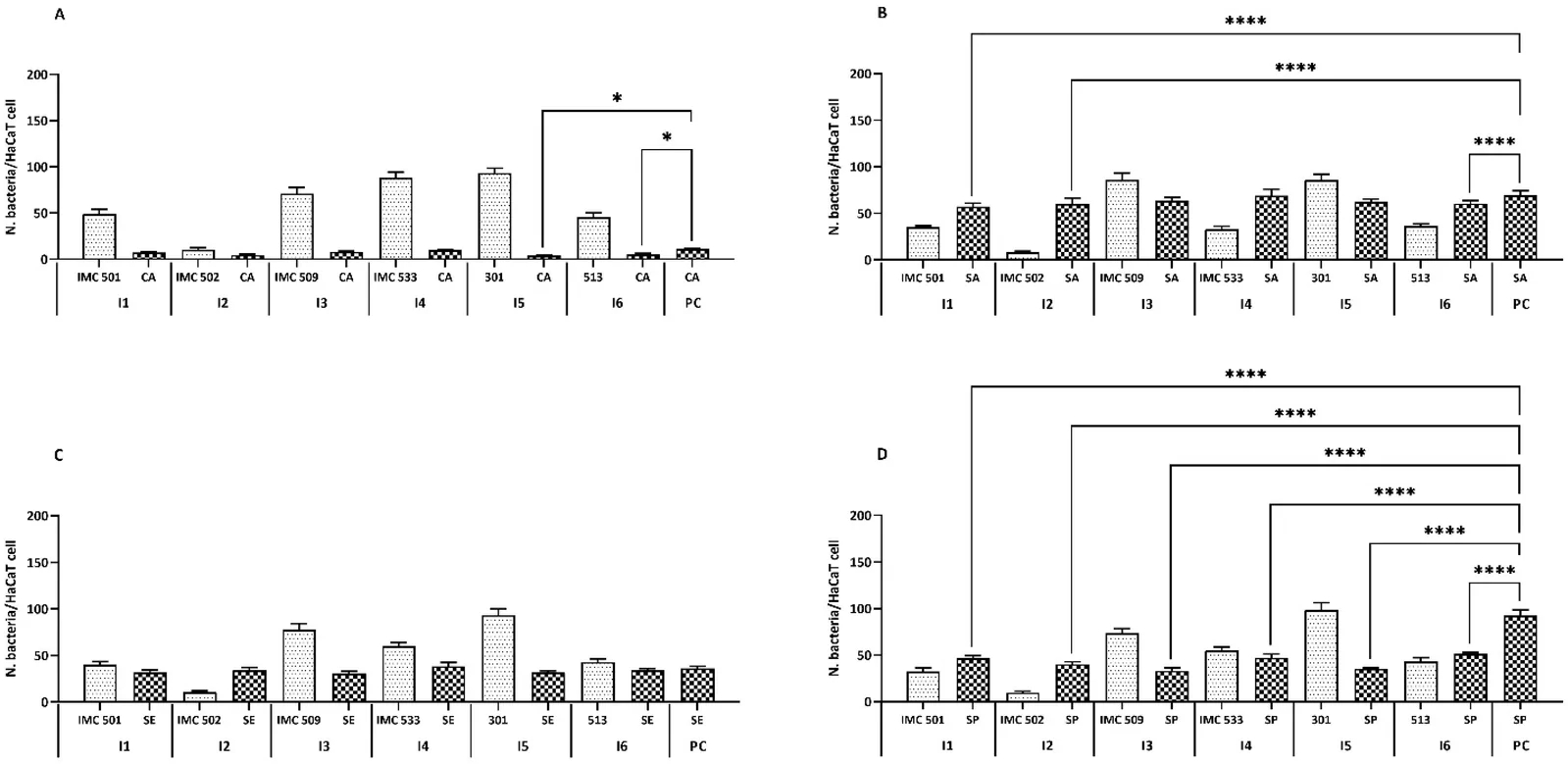
Inhibitory effect of probiotic bacteria (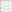) on pathogen (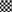) adhesion to HaCaT cells detected by inhibition assay (I1–I6). HaCaT cells were incubated in the same time with six probiotic strains (IMC 501—*L. rhamnosus* IMC 501^®^, IMC 502—*L. paracasei* IMC 502^®^, IMC 509—*L. plantarum* IMC 509, IMC 533—*L. lactis* IMC 533, 301—*L. plantarum* 301, 513—*L. rhamnosus* 513) and with CA—*Cutibacterium acnes* DSM 1897 (**A**), SP—*Streptococcus pyogenes* DSM 2071 (**B**), SE—*Streptococcus epidermidis* DSM 20044 (**C**) or SA—*Staphylococcus aureus* ATCC 25923 (**D**). Panel I1–I6 represent the six exclusion assay conditions: I1—IMC 501 vs. each pathogenic strain, I2—IMC 502 vs. each pathogenic strain, I3—IMC 509 vs. each pathogenic strain, I4—IMC 533 vs. each pathogenic strain, I5—301 vs. each pathogenic strain and I6—513 vs. each pathogenic strain. PC indicates positive control and represents the adhesion of each single pathogen. Results are expressed as mean ± SEM. Significance levels are indicated as * *p* < 0.05 and **** *p* < 0.0001 using one-way ANOVA followed by multiple comparison tests.

As Figure 4 shows, all the tested probiotic strains shown a statistically significant decrease (*p* < 0.0001) in pathogen adherence to HaCaT keratinocytes when co-incubated simultaneously (inhibition assay), with the strongest effects shown against *S. pyogenes* DSM 2071, *S. aureus* ATCC 25923 and *C. acnes* DSM 1897. The commensal *S. epidermidis* DSM 20044, on the other hand, showed moderate inhibition, indicating a microbiome-sparing impact.

### 3.4. Immune Biomarkers In Vitro Modulation

**Figure 5:**
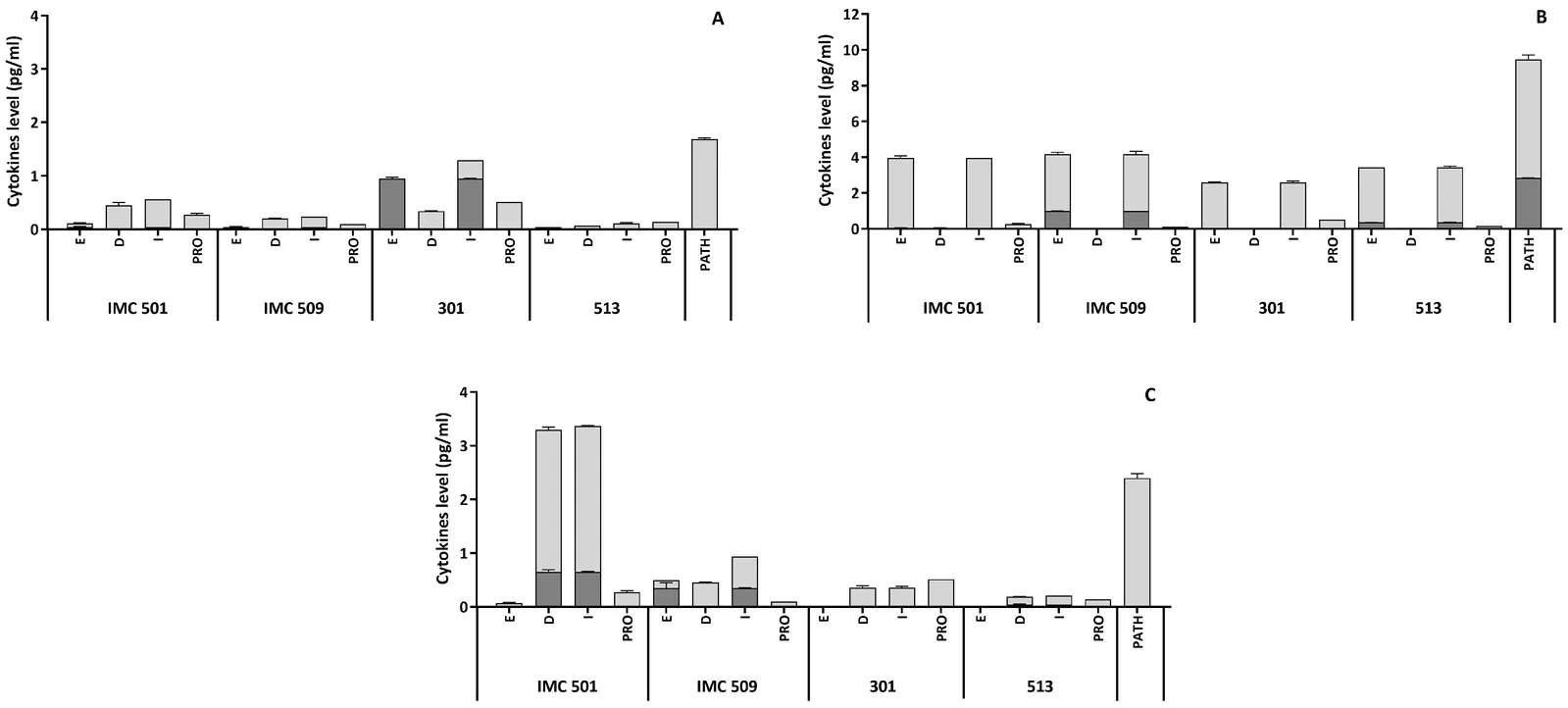
Cytokines production (IL-6 light grey bar and IL-10 dark grey bar) in HaCaT cells exposed to four probiotics (IMC 501—*L. rhamnosus* IMC 501^®^, IMC 509—*L. plantarum* IMC 509, 301—*L. plantarum* 301, 513—*L. rhamnosus* 513), and subjected to stimulation with *Cutibacterium acnes* DSM 1897 (**A**), *Staphylococcus aureus* ATCC 25923 (**B**), *Streptococcus pyogenes* DSM 2071 (**C**) by exclusion (E), displacement (D) and inhibition (I) methods compared to each probiotic (PRO) and pathogen (PATH) adhesion. Results are expressed as mean ± SD.

The ability of the probiotic strains to modulate epithelial inflammation was evaluated exposing HaCaT keratinocytes to *C. acnes* DSM 1897, *S. pyogenes* DSM 2071 and *S. aureus* ATCC 25923 under exclusion, displacement and inhibition (co-culture) conditions. Seven cytokine secretions (IFN-γ, IL-10, IL-4, IL-6, IL-8, IL-17A and TGFβ1) were quantified in all supernatants in order to provide a broad assessment of the immunoregulatory responses induced by the probiotic–pathogen interaction on HaCaT cells. Three cytokines (IFN-γ, IL-17A and TGFβ1) resulted in being below the minimum detectable concentration (MinDC), so were not included in statistical comparisons since reliable quantitative values could not be assigned, while the four cytokines, IL-6, IL-8, IL-10 and IL-4 were detected and statistically correlated with the three main acne-responsible pathogens. Figure 5 shows the levels of the higher expressed cytokines (IL-6 and IL-10) for the four probiotic strains most effective, both for antimicrobial inhibition and competition adhesion on the acne-responsible pathogens.

Across all experimental conditions, IL-6 exhibited the larger dynamic range and most noticeable modulation. Under most circumstances, IL-4 and IL-10 levels remained quite low. The exposure of HaCaT cells to *C. acnes* DSM 1897, *S. pyogenes* DSM 2071 or *S. aureus* ATCC 25923 produced pathogen-specific cytokine profiles, with IL-6 as the predominant indicator of epithelial activation. A hierarchical inflammatory response was validated by pathogen adhesion controls, with *S. aureus* ATCC 25923 causing the highest amounts of IL-6, followed by *S. pyogenes* DSM 2071 and *C. acnes* DSM 1897. Under exclusion conditions, several probiotic strains significantly attenuated IL-6 secretion compared to that with pathogen adhesion alone. The strain 301 demonstrated the most consistent anti-inflammatory effect across all pathogens, reducing IL-6 to near-baseline levels for *C. acnes* DSM 1897 and *S. pyogenes* DSM 2071, and partially attenuating *S. aureus* ATCC 25923-induced activation. Moreover, both *L. plantarum* strains, IMC 509 and 513, also exhibited protective profiles. The combined evaluation of IL-6, as a marker of pro-inflammatory activation, and IL-10, as an immunoregulatory cytokine, allowed the characterization of the predominant inflammatory versus regulatory response associated with the most effective probiotic strains.

On the other hand, displacement and inhibition assays showed significant strain-dependent variability. Generally, IL-8 changing levels were similar to those of IL-6, however, they were lower. IL-10 induction was mild and mostly seen with strain 301, whereas IL-4 remains close to the detection limit. These findings document that probiotic-mediated modulation of keratinocyte inflammation is strongly strain specific and most effective under preventive conditions (exclusion).

## 4. Discussion

Finding effective novel therapies for acne, an inflammatory skin condition, was the aim of the current research. Evidence suggests that probiotics may help decrease skin eruptions, also highlighting the role of the gut microbiota that plays in the development of acne lesions [37,38]. The gut microbiota is essential to maintain a healthy immune system. Therefore, acne may be related to the state of the gastrointestinal tract and its microbial balance. Due to their rapid growth, the combination of probiotic dietary supplements and probiotic-based “cosmetics” has enormous potential for improving skin health in acne-affected persons.

The present study establishes that the six probiotic strains possess an anti-acne potential, integrating antimicrobial activity, epithelial adhesion, competitive exclusion of pathogens and immunomodulatory properties in keratinocyte models. These effects were found to be highly strain dependent, underscoring the relevance of targeted probiotic selection for dermatological applications.

Regarding the antimicrobial activity, both radial streak and agar well diffusion assays confirmed that all tested probiotic strains inhibited key skin-associated pathogens, including *C. acnes* DSM 1897, *S. aureus* ATCC 25923, *S. pyogenes* DSM 2071 and *S. epidermidis* DSM 20044. However, both the extent and spectrum of inhibition varied considerably among strains. In particular, both strains belonging to *L. rhamnosus* (IMC 501^®^ and 513) and *L. plantarum* (IMC 509 and 301) exhibited the most consistent and antimicrobial broad-spectrum, whereas *L. lactis* IMC 533 showed weaker effects. These findings are in agreement with previous studies indicating that *L. rhamnosus* and *L. plantarum* are capable of producing several antimicrobial compounds, including organic acids, bacteriocins and biosurfactants, which may contribute to the pathogenic inhibition [5,6,39]. The radial streak method showed, generally, stronger and more discriminative antimicrobial activity compared to the agar well diffusion assay, suggesting that direct cell-to-cell interactions and localized metabolite production play a major role in pathogen inhibition. On the other hand, the results obtained from agar well diffusion assays suggest that cell-free supernatant retain substantial antimicrobial activity, demonstrating that inhibition is primarily mediated by secreted metabolites rather than direct bacterial contact. This observation supports the growing evidence that postbiotic compounds are playing a key role in mediating probiotic effects in skin disorders [39,40]. Among the tested pathogens, *S. pyogenes* DSM 2071 was the most susceptible, whereas *C. acnes* DSM 1897 exhibited relatively lower sensitivity. This diminished susceptibility may be attributed to the anaerobic nature and follicular niche adaptation of *C. acnes*, which can limit exposure to diffusible antimicrobial compounds [41].

On the other side, adhesion to keratinocytes represents a critical prerequisite for probiotic colonization and functional activity on the skin. All probiotic strains demonstrated the ability to adhere to HaCaT cells, with *L. plantarum* 301 showing the highest adhesion efficiency, followed by IMC 509. These findings are confirmed by previous studies highlighting the role of bacterial surface components (such as proteins, lipoteichoic acids and exopolysaccharides) in mediating adhesion to epithelial cells [42]. Notably, probiotic strains generally exhibited higher adhesion capacity compared to pathogenic bacteria, suggesting a potential competitive advantage in colonizing the skin. Nevertheless, some discrepancies between viable bacterial counts and microscopic observations (e.g., IMC 533) indicate that adhesion is a complex and multifactorial process, possibly influenced by bacterial aggregation or methodological variability.

Moreover, competition assays provided further mechanistic insight into probiotic–pathogen interactions at the epithelial interface. In exclusion assays, pre-colonization of keratinocytes with probiotics significantly decreased pathogen adhesion across all tested species, supporting the concept that probiotics can function as a preventive barrier by occupying binding sites and limiting pathogen access. In addition, substantial scientific evidence [5,6] has shown that this mechanism is widely recognized in mucosal microbiology and deeply described for skin ecosystems. In contrast, displacement assays revealed a more limited efficacy, indicating that probiotics are generally less capable of removing already-adhered pathogens which exhibit resistance to displacement, as also observed by Costello and co-workers [43]. Nonetheless, two probiotic strains (IMC 502 and IMC 533) demonstrated measurable displacement capacity, suggesting potentially therapeutic relevance in already colonized environments. Probiotic–pathogen co-incubation (inhibition) assays confirmed strong competitive interactions during early colonization stages, with significant reductions in pathogen adhesion. Notably, *S. epidermidis* DSM 20044, a key skin commensal, was less affected, indicating a microbiome-sparing effect, which is particularly advantageous in the context of acne control. Removing adhered pathogenic bacteria is more difficult than preventing initial colonization, as demonstrated by the displacement assay, which showed a lower but still significant anti-adhesive impact of probiotic strains when compared to the exclusion assay. Interestingly, two probiotic strains, IMC 502 and IMC 533, were able to successfully remove *C. acnes* DSM 1897 and *S. pyogenes* DSM 2071 from HaCaT cells, while they only partially removed the commensal *S. epidermidis* DSM 20044, indicating microbiome-selective action. The strain-dependent effectiveness suggests that competitive surface contacts and probiotic adhesion strength are crucial for post-adhesion pathogen control.

Overall, these findings suggest that probiotics primarily act through preventive ecological mechanisms, rather than direct pathogen eradication, reinforcing their potential role in long-term microbiome modulation strategies.

Although both adhesion and competition assays performed on HaCaT cells provide valuable mechanistic insights on the adhesion ability of probiotic strains to epithelial cells and interfere with pathogens’ colonization, they represent a simplified model of the human skin environment. In vivo, bacterial colonization is influenced by several factors, such as multi-layered epidermal structure, stratum corneum presence, sebum and sweat components, resident skin microbiome, immune cells and dynamic physicochemical conditions, not fully reproduced in vitro. Therefore, the present findings should be considered as evidence of the probiotic strains’ potential to establish early interactions with the skin epithelium through adhesion capacity and competitively limit pathogens’ adhesion, rather than as direct predictors of long-term skin colonization or clinical efficacy. Further validation using reconstructed human skin models and/or clinical studies is warranted to confirm these effects under physiological conditions.

Another key outcome of this in vitro study was the ability of probiotic strains to modulate keratinocyte inflammatory responses, mainly through the IL-6 modulation. Pathogenic exposure induced an inflammatory response, with *S. aureus* ATCC 25923 triggering the highest IL-6 production, followed by *C. acnes* DSM 1897 and *S. pyogenes* DSM 2071, consistent with previous observations of pathogen-specific immune activation [44]. Under exclusion conditions, several probiotic strains, most notably *L. plantarum* 301, significantly decreased IL-6 secretion, in some cases restoring levels close to baseline. This suggests that probiotics may prevent pathogen-induced inflammation, likely through interference with adhesion processes and modulation of epithelial signaling pathways, as also suggested by Kim [23]. On the contrary, certain strains (e.g., *L. rhamnosus* IMC 502^®^) enhanced IL-6 production under co-culture conditions, highlighting that probiotic effects are not universally beneficial and depend on strain-specific host interactions. IL-10 levels remained low, evidencing that the predominant effect is the attenuation of pro-inflammatory signaling rather than the induction of anti-inflammatory cytokines, in line with the reported data by Łętocha and co-workers [45].

All these results support a multimodal function of probiotic-mediated inhibition of skin pathogens and modulation of innate immune responses in HaCaT cells. This includes direct antimicrobial action against skin pathogens; competitive exclusion mechanisms which interfere with pathogen colonization; selective modulation of the skin microbiota while maintaining commensals; inflammation decrease, especially through IL-6-related pathways.

It is well known that probiotic efficacy cannot be generalized and the higher performance of *L. rhamnosus* and *L. plantarum* strains tested highlights the significance of strain-specific selection. Furthermore, these probiotic strains may be especially useful as preventative or maintenance measures rather than as stand-alone therapies for existing skin issues, as evidenced by the higher efficacy observed in exclusion assays in respect to displacement assays. The topical probiotic effects on skin may also be strongly supported by an oral probiotic supplementation which modulates acne pathophysiology through interconnected mechanisms involving the gut–skin axis balancing the intestinal microbiota and controlling the inflammatory profile.

On the other hand, the use of pathogenic bacteria was needed to explore specific probiotic–pathogen interactions, and their direct therapeutic use is not envisaged. Instead, these findings provide a mechanistic basis for developing targeted narrow-spectrum antimicrobials or postbiotic preparations that selectively target pathogens while preserving host tissues and the commensal microbiota. Future translational applications should prioritize non-viable microbial components or properly attenuated strains, developed under appropriate biosafety conditions.

## 5. Conclusions

In conclusion, the present investigation documents that some probiotic strains, belonging to *L. rhamnosus* and *L. plantarum*, exhibit significant anti-acne potential through a combination of antimicrobial, competitive and immunomodulatory mechanisms. These findings support the concept that probiotics are specific modulators of skin microbiota, offering a promising complementary strategy for managing and preventing acne. Further validation in more physiologically relevant skin models and in vivo studies is therefore required to confirm the reproducibility, mechanisms of action, safety and potential therapeutic applicability of these probiotic candidates.

## Figures and Tables

**Table 1:** Bacterial strains used in the study and their growth conditions.

**Probiotic Strain ^1^**	**Strain Code**	**Growth Conditions**
*Lacticaseibacillus rhamnosus* IMC 501^®^	IMC 501	MRS ^2^, 48 h at 37 °C
*Lacticaseibacillus paracasei* IMC 502^®^	IMC 502	MRS, 48 h at 37 °C
*Lactiplantibacillus plantarum* IMC 509	IMC 509	MRS, 48 h at 37 °C
*Lactococcus lactis* IMC 533	IMC 533	MRS, 48 h at 37 °C
*Lactiplantibacillus plantarum* 301	301	MRS, 48 h at 37 °C
*Lacticaseibacillus rhamnosus* 513	513	MRS, 48 h at 37 °C
**Pathogenic Strains**	**Strain Code**	**Growth Conditions**
*Cutibacterium acnes* DSM 1897	CA	TSB ^3^ 24 h at 37 °C
*Staphylococcus aureus* ATCC 25923	SA	TSB 24 h at 37 °C
*Staphylococcus epidermidis* DSM 20044	SE	TSB 24 h at 37 °C
*Streptococcus pyogenes* DSM 2071	SP	TSB 24 h at 37 °C

^1^ Synbiotec Srl, Camerino (MC), Italy; ^2^ MRS-de Man, Rogosa, Sharpe broth (OXOID); ^3^ TSB-Tryptone Soy Broth (OXOID); DSM: German Collection of Microorganisms and Cell Cultures; ATCC: American Type Culture Collection.

**Table 2:** Minimum detectable concentrations (MinDC) for cytokines (pg/mL).

Cytokines	MinDC (pg/mL)
IFNγ	0.86
IL-4	0.20
IL-6	0.14
IL-8	0.52
IL-10	0.91
IL-17A	0.71
TGF-β1	6.00

**Table 3:** Inhibition zone diameter (mm) produced by each CFS of probiotic strain cultures against the pathogens tested by agar well diffusion assay.

CFS of Probiotic Strains	Inhibition Zone (mm) *			
	*C. acnes* DSM 1897	*S. aureus* ATCC 25923	*S. epidermidis* DSM 20044	*S. pyogenes* DSM 2071
***L. rhamnosus*** **IMC 501^®^**	11.8 ± 0.6 ^a^	17.9 ± 0.8 ^a^	13.2 ± 0.9 ^ab^	21.6 ± 1.3 ^a^
***L. paracasei*** **IMC 502^®^**	11.1 ± 0.9 ^a^	16.7 ± 1.1 ^ab^	12.2 ± 0.9 ^ab^	19.1 ± 0.4 ^ab^
***L. plantarum*** **IMC 509**	11.5 ± 1.3 ^a^	14.7 ± 1.8 ^b^	14.9 ± 0.6 ^a^	19.5 ± 0.9 ^ab^
***L. lactis*** **IMC 533**	5.7 ± 0.6 ^b^	9.9 ± 0.6 ^c^	10.7 ± 3.04 ^b^	17.9 ± 1.2 ^b^
***L. plantarum*** **301**	11.5 ± 1.1 ^a^	14.0 ± 1.1 ^b^	14.0 ± 1.0 ^ab^	19.5 ± 0.9 ^ab^
***L. rhamnosus*** **513**	10.4 ± 0.6 ^a^	16.2 ± 0.3 ^ab^	15.5 ± 1.4 ^a^	19.6 ± 0.3 ^ab^

* Mean values ± standard deviation (SD) of three determinations (n = 3); different letters within the same column indicate statistically significant differences (*p* < 0.05) by one-way ANOVA followed by Tukey’s post hoc test.

**Table 4:** Inhibition zone (mm) produced by each probiotic strain against the pathogens tested by radial streak method.

Probiotic Strains	Inhibition Zone (mm) *			
	*C. acnes* DSM 1897	*S. aureus* ATCC 25923	*S. epidermidis* DSM 20044	*S. pyogenes* DSM 2071
***L. rhamnosus*** **IMC 501^®^**	6.9 ± 1.6 ^a^	12.6 ± 1.3 ^ab^	18.7 ± 0.2 ^a^	17.1 ± 0.6 ^a^
***L. paracasei*** **IMC 502^®^**	5.4 ± 0.4 ^ab^	10.1 ± 0.9 ^b^	16.0 ± 0.7 ^b^	12.0 ± 1.9 ^b^
***L. plantarum*** **IMC 509**	8.6 ± 1.6 ^a^	14.5 ± 0.6 ^a^	20.0 ± 0.6 ^a^	13.8 ± 1.7 ^ab^
***L. lactis*** **IMC 533**	2.3 ± 1.8 ^b^	8.8 ± 1.6 ^b^	13.7 ± 0.5 ^c^	5.4 ± 0.8 ^c^
***L. plantarum*** **301**	8.2 ± 1.3 ^a^	12.9 ± 1.6 ^ab^	20.2 ± 0.4 ^a^	13.2 ± 1.4 ^b^
***L. rhamnosus*** **513**	7.7 ± 0.9 ^a^	12.1 ± 1.2 ^ab^	19.3 ± 0.8 ^a^	14.6 ± 0.8 ^ab^

* Mean values ± standard deviation (SD) of three determinations (n = 3); different letters within the same column indicate statistically significant differences (*p* < 0.05) by one-way ANOVA followed by Tukey’s post hoc test.

**Table 5:** Adhesion capacity of bacterial strains to HaCaT cells, evaluated by viable cell counting (CFU/mL) and by direct bacteria counting in light microscope fields on Gram-stained HaCaT cells.

Strains	Bacterial Cell Count (CFU/mL)		Adhesion Percentage * (%)	Number of Adherent Bacteria per Cell ^#^	Adhesion Index ^§^
	Initial (T0)	Adhered Bacteria (T1)			
**PROBIOTICS**					
***L. rhamnosus*** **IMC 501^®^**	(1.6 ± 0.1) × 10^9^	(6.9 ± 0.6) × 10^7^	4.5	43.6 ± 24.8	1334
***L. paracasei*** **IMC 502^®^**	(2.5 ± 0.0) × 10^9^	(1.1 ± 0.0) × 10^8^	4.4	12.9 ± 7.4	502
***L. plantarum*** **IMC 509**	(4.2 ± 0.1) × 10^9^	(2.7 ± 0.0) × 10^8^	6.5	73.6 ± 32.8	4212
***L. lactis*** **IMC 533**	(7.1 ± 0.2) × 10^8^	(2.4 ± 0.0) × 10^7^	3.4	98.3 ± 47.0	4467
***L. plantarum*** **301**	(2.5 ± 0.2) × 10^9^	(2.8 ± 0.1) × 10^8^	11.1	63.5 ± 25.2	4160
***L. rhamnosus*** **513**	(3.2 ± 0.1) × 10^9^	(1.2 ± 0.0) × 10^8^	3.9	50.4 ± 23.4	2761
**PATHOGENS**					
***C. acnes*** **DSM 1897**	(1.3 ± 0.2) × 10^9^	(1.1 ± 0.1) × 10^7^	0.9	11.02 ± 5.73	238
***S. aureus*** **ATCC 25923**	(1.0 ± 0.1) × 10^9^	(3.0 ± 0.1) × 10^7^	2.9	69.0 ± 37.6	3510
***S. epidermidis*** **DSM 20044**	(1.2 ± 0.1) × 10^8^	(2.5 ± 0.4) × 10^6^	2.1	35.9 ± 18.2	2204
***S. pyogenes*** **DSM 2071**	(5.5 ± 0.2) × 10^9^	(1.9 ± 0.2) × 10^8^	3.3	92.2 ± 42.1	4694

* Adhesion percentage calculated on bacterial cell counts adhered to HT29 cells from the total number of bacteria added (CFU/mL); ^#^ calculated evaluating 50 cells randomly; mean values ± standard deviation of two determinations; ^§^ bacteria adhering per 100 HaCaT cells evaluating twenty microscopic fields.

## Data Availability

The original contributions presented in this study are included in the article/Supplementary Materials. Further inquiries can be directed to the corresponding author.

## Supplementary Materials

The following supporting information can be downloaded at https://www.mdpi.com/article/10.3390/microorganisms14081754/s1: Table S1. Three-way factorial analysis of pathogen adhesion.
