# Supplementary material for: Probiotic-Mediated Inhibition of Skin Pathogens and Modulation of Innate Immune Responses in HaCaT Cells
Source: Microorganisms. 2026 Aug 10;14(8):1754. doi: 10.3390/microorganisms14081754 (PMC13515721; doi:10.3390/microorganisms14081754)
Supplement: Supplementary file 1 [file microorganisms-14-01754-s001.zip › microorganisms-4386178-supplementary.pdf]

Table S1. Three-way factorial analysis of pathogen adhesion.

Results of a Type III three-way factorial ANOVA evaluating the effects of probiotic strain, pathogen species, competition assay, and their interactions on the number of adhered pathogenic bacteria. Experimental replicate was included as a blocking factor.

Effect	df*	F	P value	Partial η^2	95% CI for partial η^2
Replicate (blocking factor)	2	181.97	< 0.0001	0.687	0.612–0.742
Probiotic strain	6	198.15	< 0.0001	0.877	0.846–0.900
Pathogen	3	2948.51	< 0.0001	0.982	0.977–0.985
Competition assay	2	313.21	< 0.0001	0.791	0.739–0.829
Strain × Pathogen	18	94.49	< 0.0001	0.911	0.887–0.927
Strain × Assay	12	53.06	< 0.0001	0.793	0.738–0.829
Pathogen × Assay	6	96.16	< 0.0001	0.777	0.720–0.816
Strain × Pathogen × Assay	36	27.65	< 0.0001	0.857	0.814–0.880

*df are reported as numerator and denominator degrees of freedom. Partial η^2 was calculated as SS (Sum of Squares) effect/(SS effect + SS error). Confidence intervals were obtained by inversion of the noncentral F distribution. All main effects and interaction terms were statistically significant. The significant three-way interaction indicates that the effect of probiotic strain on pathogen adhesion varies according to both pathogen species and competition assay (exclusion, displacement, or inhibition).
